# The genome of Chinese flowering cherry (*Cerasus serrulata*) provides new insights into *Cerasus* species

**DOI:** 10.1038/s41438-020-00382-1

**Published:** 2020-10-01

**Authors:** Xian-Gui Yi, Xia-Qing Yu, Jie Chen, Min Zhang, Shao-Wei Liu, Hong Zhu, Meng Li, Yi-Fan Duan, Lin Chen, Lei Wu, Shun Zhu, Zhong-Shuai Sun, Xin-Hong Liu, Xian-Rong Wang

**Affiliations:** 1 Co-Innovation Center for the Sustainable Forestry in Southern China, College of Biology and the Environment; Cerasus Research Center, Nanjing Forestry University, 210037 Nanjing, Jiangsu China; 2 College of Horticulture, Nanjing Agricultural University, 210095 Nanjing, Jiangsu China; 3 College of Food Science and Technology, Nanjing Agricultural University, 210095 Nanjing, Jiangsu China; 4 Biomarker Technologies Corporation, 101300 Beijing, China; 5 Zhejiang Provincial Key Laboratory of Plant Evolutionary Ecology and Conservation, Taizhou University, 318000 Taizhou, Zhejiang China; 6 Zhejiang Academy of Forestry, 310023 Hangzhou, Zhejiang China

**Keywords:** Genome assembly algorithms, Genetic variation, Comparative genomics

## Abstract

*Cerasus serrulata* is a flowering cherry germplasm resource for ornamental purposes. In this work, we present a de novo chromosome-scale genome assembly of *C. serrulata* by the use of Nanopore and Hi-C sequencing technologies. The assembled *C. serrulata* genome is 265.40 Mb across 304 contigs and 67 scaffolds, with a contig N50 of 1.56 Mb and a scaffold N50 of 31.12 Mb. It contains 29,094 coding genes, 27,611 (94.90%) of which are annotated in at least one functional database. Synteny analysis indicated that *C. serrulata* and *C. avium* have 333 syntenic blocks composed of 14,072 genes. Blocks on chromosome 01 of *C. serrulata* are distributed on all chromosomes of *C. avium*, implying that chromosome 01 is the most ancient or active of the chromosomes. The comparative genomic analysis confirmed that *C. serrulata* has 740 expanded gene families, 1031 contracted gene families, and 228 rapidly evolving gene families. By the use of 656 single-copy orthologs, a phylogenetic tree composed of 10 species was constructed. The present *C. serrulata* species diverged from *Prunus yedoensis* ~17.34 million years ago (Mya), while the divergence of *C. serrulata* and *C. avium* was estimated to have occurred ∼21.44 Mya. In addition, a total of 148 MADS-box family gene members were identified in *C. serrulata*, accompanying the loss of the AGL32 subfamily and the expansion of the SVP subfamily. The MYB and WRKY gene families comprising 372 and 66 genes could be divided into seven and eight subfamilies in *C. serrulata*, respectively, based on clustering analysis. Nine hundred forty-one plant disease-resistance genes (R-genes) were detected by searching *C. serrulata* within the PRGdb. This research provides high-quality genomic information about *C. serrulata* as well as insights into the evolutionary history of *Cerasus* species.

## Introduction

**Fig. 1:**
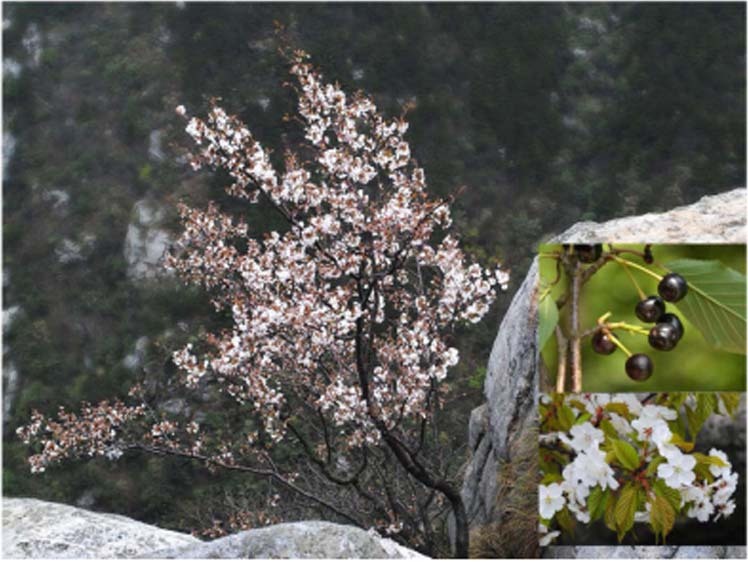
Images of a Chinese flowering cherry tree and its flowers and berries from March to April 2019. This Chinese flowering cherry tree grows in Baohua mountain, Jiangsu Province. The lower right corner shows a close-up of cherry flowers and berries

*Cerasus serrulata* (Lindley) Loudon belongs to *Cerasus* Mill. in the Rosaceae family^1,2^. The corymbose-racemose or subumbellate inflorescences of this species usually have 2–3 (5) flowers, and most flowers are white or pale pink, with single petals^3^. Trees of *C. serrulata* (*Cerasus serrulata*) have dark green leaves with acuminate serrate teeth on the margins, dense flowers with unfolding petals, and black fruits^4,5^ (Fig. 1). *Cerasus serrulata* is the parent of many ornamental cherry varieties and is an important flowering cherry germplasm resource used for ornamental purposes^6^.

*Cerasus serrulata* is widely distributed in the midwestern and eastern regions of China and possibly also is present on the Korean Peninsula and in Japan^3,5^. China has the most abundant germplasm resources of *C. serrulata*, where various natural and artificial hybrids have been developed and selected^7,8^. More than 45 species (31 endemics) and nine varieties occur in Southwest China, along the middle and lower reaches of the Yangtze River, in Northeast China, and in Chinese Taipei (there are ~16 species and 11 varieties in Japan)^4,9^. Due to a long history of cultivation along with the naturalization of wild flowering cherry species and interspecific hybridization in China, there has been much taxonomic debate over the name, origin, and delimitation among different populations in *C. serrulata* (especially different populations in alpine areas). Numerous investigations into *C. serrulata*, including the collection of specimens, development of morphological markers and molecular markers, and assessment of evolutionary and phylogeographic relationships, have been carried out to determine its origin^10,11^. The interpretation of Li Chao-Luang and Bruce Bartholomew in the Flora of China was adopted here in dealing with taxonomic disputes^4^. Considering that genomics-based research could be fundamentally helpful in overcoming this drawback to some extent, a de novo genome sequencing project was launched in this study, aiming to provide a scientific and theoretical basis for understanding *C. serrulata*.

The acquisition of a high-quality genome may provide more detailed insights into the evolutionary history and contribute toward the settlement of disputes over the classification. In the past decade, whole-genome sequencing has been widely performed on a number of Rosaceae species, including the fruit crop species sweet cherry^12^, peach^13^, apple^14^, Chinese plum^15^, strawberry^16^, apricot^17^, and black raspberry^18^, as well as the ornamental species *Prunus* x *yedoensis*^19^, plum blossom^20^ Chines rose^21^, and multiflora rose^22^. In this study, we successfully assembled the genome of *C. serrulata* (2*n* = 2*x* = 16) and compared it with the genomes of closely related species in terms of the gene family, positive gene selection, and phylogeny. This work provides a foundation for clarifying the genetic variation, genetic diversity, and genealogical structure of *C. serrulata*.

## Results

### Genome estimation and assembly

The genome size of *C. serrulata* was estimated by *k*-mer analysis to be 256.65 Mb with repeat and heterozygosity percentages of 46.55% and 1.67%, respectively (Supplementary Fig. S1). In this work, 1.88 million clean reads (~47.99 Gb) were obtained, with a read N50 of 32.08 bb and a read mean length of 25.49 bb (Table 1). The results of BLAST with randomly selected reads aligned to the nucleotide sequence database (Nt) were used to assess contamination. A total of 43.60% and 25.59% of mapped reads on the alignment were represented by *P. mume* and *P. persica*, respectively (Supplementary Fig. S2). A Nanopore long-read distribution was computed using different gradient lengths (Supplementary Table S1). Primary assembly was employed by Canu v1.5, WTDBG v1.1, and SMARTdenovo, with adjusted parameters, resulting in an optimized primary assembled genome of 265.38 Mb distributed across 182 contigs.

**Table 1:** Statistics and characteristics of the genome of *C. serrulata*

Characteristics	Number	Size	Sequence coverage (X)	Percentage
Illumina reads		38.14 Gb	148.62	
Nanopore reads		47.99 Gb	180.84	
Hi-C reads		36.77 Gb	143.28	
The estimate of genome size		256.65 Mb		
Final-assembly genome size		265.40 Mb		
Contig number and N50	304	1.56 Mb		
Maximum contig		7.34 Mb		
Scaffold number and N50	67	31.12 Mb		
Maximum scaffold		49.87 Mb		
GC content				38.51%
Heterozygosity percentage				1.67%
Total repetitive sequences		130.11 Mb		49.02%
Total protein-coding genes	29,094	104.75 Mb		
Annotated protein-coding genes	27,611			94.90%
MicroRNA	94			
Ribosomal RNA (rRNA)	315			
Transfer RNA (tRNA)	472			

In total, 122.84 million paired reads were generated from Hi-C, of which 95.72 million paired reads (77.92%) were mapped to the primary assembly, and 53.35 million paired reads (43.43%) were uniquely mapped. Furthermore, 41.20 million valid interaction pairs were strongly mapped to the unique mapped paired reads, accounting for 77.23% (Supplementary Table S2). A total of 182 primary contigs were fragmented and reassembled using the Lachesis combined Hi-C data. A total of 263.16 Mb genomic sequences, accounting for 99.16% of total contig sequences, were located. Two hundred and eighty-nine corrected contigs were clustered into eight groups using the agglomerative hierarchical clustering method. Among them, 245 ordered contigs (252.25 Mb) were anchored with a defined order and orientation (Supplementary Table S3).

**Fig. 2:**
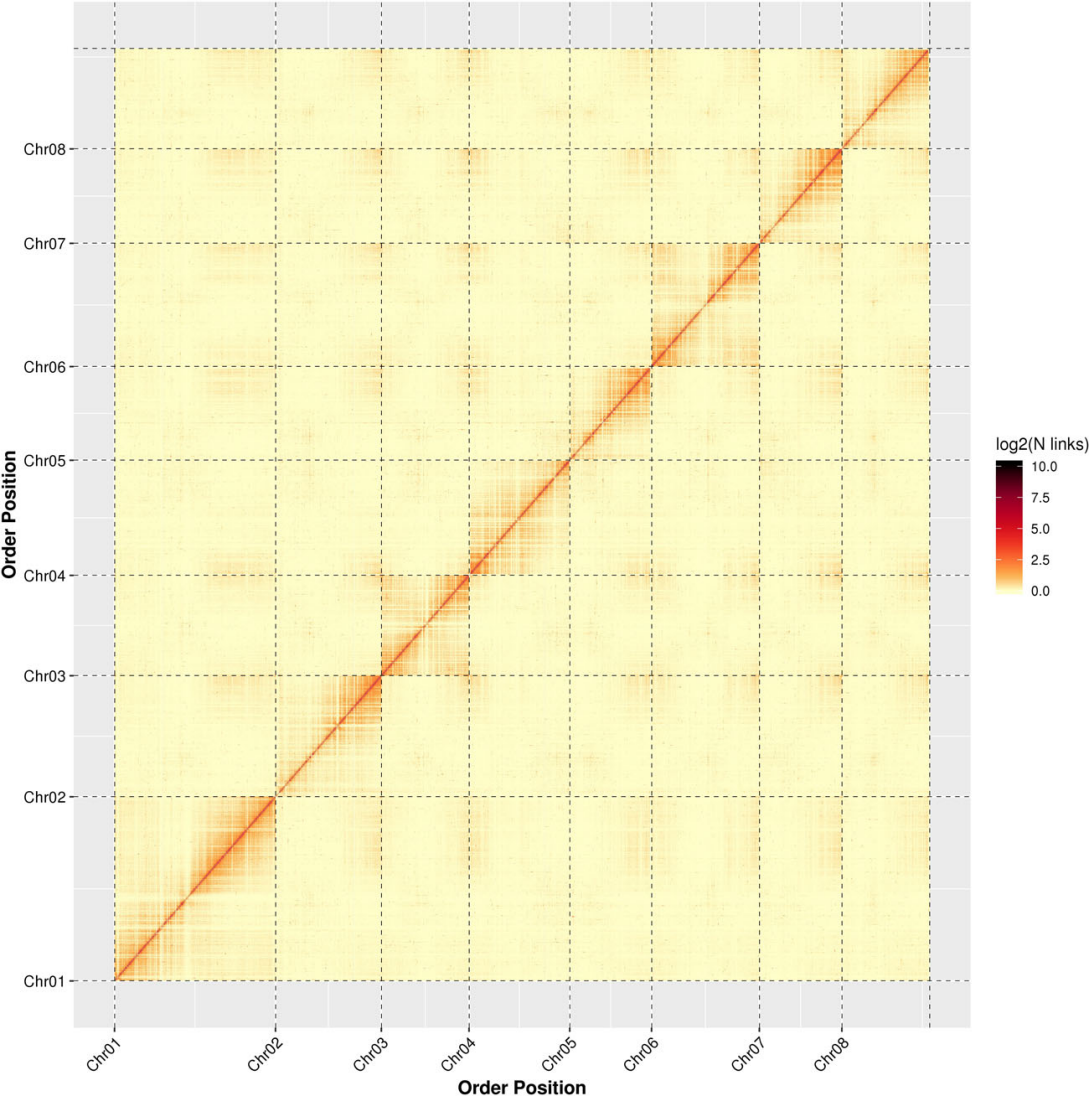
Hi-C interaction heatmap for *Cerasus serrulata.* Chr01–08 are the abbreviations of chromosome 1–8

The final unambiguous chromosomal-level genome of *C. serrulata* with no obvious assembly errors was composed of eight clusters, as indicated in the Hi-C interaction heatmap (Fig. 2). The chromosomal genome of *C. serrulata* is 265.40 Mb in length and is characterized by 304 contigs and 67 scaffolds, with a contig N50 of 1.56 Mb and a scaffold N50 of 31.12 Mb (Table 1).

### Genome assessment and annotation

BUSCO analysis showed that, of the 1614 expected embryophytic genes (embryophyta_odb10), 1528 (94.67%) complete BUSCO genes were identified, and 1352 genes were complete single-copy genes (Supplementary Table S4). CEGMA analysis indicated that of the 458 conserved core genes in eukaryotes, 447 (97.60%) core genes were identified based on sequence similarity (Supplementary Table S4). Approximately 254.77 million reads (98.08%) for Illumina were mapped to the assembly, and the number of mapped paired reads was ~230.62 million reads (88.79%) (Supplementary Table S4).

A high degree of consistency among the BUSCO and CEGMA data and the mapping ratio for Illumina confirms the high quality of the assembled genome. The repetitive sequences were 130.11 Mb in total length, representing 49.02% of the genome (Table 1). Approximately 63.20 Mb of long terminal repeats (LTRs), representing 48.57% of all repeat types, were dominant. *Copia* (25.27 Mb) and *Gypsy* (34.23 Mb) were the most abundant subtypes, representing 19.42% and 26.31%, respectively. The characteristics of the repeats, including the number, length, and percentage of each repeat type, were captured in detail (Supplementary Table S5).

Ab initio-, homology-based-, and transcriptome-based predictions were achieved using various workflows (Supplementary Table S6). The results of the predictions were integrated into the final nonredundant protein-coding genes using EVM (Table 1). These 29,094 (104.75 Mb) coding genes were composed of 157,063 exons, with an average gene length of 3.61 kb. For genome annotation, 27,611 genes, accounting for 94.90% of predicted coding genes, were annotated in at least one functional database. Gene annotation numbers and percentages were calculated by aligning to all the databases (Supplementary Table S7), and each gene annotation is provided in great detail in Supplementary Table S8. In addition, the *C. serrulata* genome was characterized by 2170 pseudogenes, 94 microRNAs, 315 rRNAs, and 472 tRNAs (Supplementary Table S9).

### Synteny analysis between *C. serrulata* and both *P. persica* and *C. avium*

**Fig. 3:**
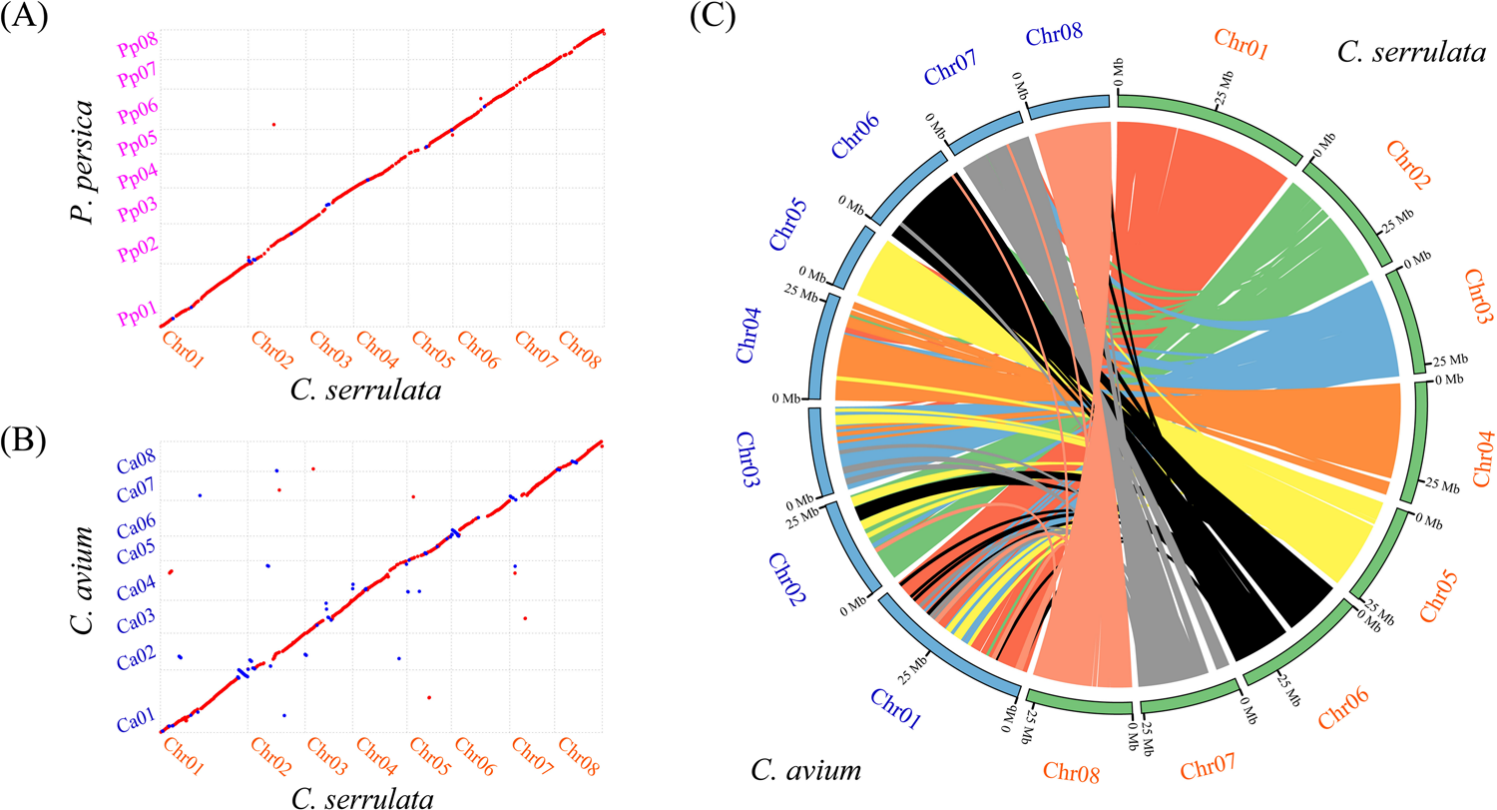
Synteny analysis via comparisons of *Cerasus serrulata* with *Prunus persica* and *Cerasus avium*. **a** Colinear map of the *C. serrulata* genome and *P. persica* genome. The red color represents identical sequences in the same orientation, while the blue color represents identical sequences in the opposite orientation. **b** Colinear map of the *C. serrulata* genome and *C. avium* genome. The red color represents identical sequences in the same orientation, while the blue color represents identical sequences in the opposite orientation. **c** Syntenic blocks of *C. serrulata* and *C. avium*

Two highly consistent colinear maps were constructed by comparing the *C. serrulata* genome with the *P. persica* and *C. avium* genomes. The colinear map indicated that the sequences of the *C. serrulata* genome were practically identical to those of the *P. persica* genome, as indicated by the red lines on the diagonal (Fig. 3a). Only a few spots of blue color represent identical sequences of opposite orientation between the two genomes. Interestingly, the colinear map of *C. serrulata* and *C. avium* showed that they possessed numerous identical sequences on different chromosomes (Fig. 3b). Among their differences, identical sequences in opposite orientations constituted a relatively high proportion. The map indicated that, compared with the *P. persica* genome, the *C. serrulata* genome is of extraordinarily high quality. The spots on the colinear map may exist and might have caused some functional changes between the two species or otherwise could have resulted from assembly errors. The syntenic blocks generated by comparing the *C. avium* genome with the *C. serrulata* genome were distributed across eight similar chromosomes (Fig. 3c). We obtained 333 syntenic blocks, which were composed of 14,072 genes, across the two genomes (Supplementary Tables S10 and S11). Among the 333 syntenic blocks, there are 193 common blocks with an accurate match on the same chromosome, representing 57.96% of the total blocks. Thirty-six blocks on chromosome 04 of *C. serrulata* were discovered in *C. avium*, of which 33 occurred on chromosome 04 of *C. avium*. The results also indicated that 58 blocks on chromosome 01 of *C. serrulata* were distributed on all of the chromosomes of *C. avium* and were not found on other chromosomes in *C. serrulata*. These findings imply that chromosome 04 of *C. serrulata* is relatively conserved, while chromosome 01 is the most ancient or has been the most active chromosome throughout evolution.

### Evolutionary analysis of *C. serrulata*

Based on the combined results from BLASTP and Pfam searches, gene families were identified and subjected to comparative genomic analysis to reveal their evolutionary relationships. The results revealed that there were 14,792 gene families in the *C. serrulata* genome and that clustered with the annotated 29,094 protein-coding genes. Among the 14,792 gene families, 4451 gene families were unique or specific to the *C. serrulata* genomes compared with the genomes of *C. avium* and *P. yedoensis* (Supplementary Fig. S3a). The 4451 gene families clustered with 5671 genes annotated by Pfam and other databases (Supplementary Table S12). The enrichment results for GO functional analysis involved mainly transmembrane transport, endonuclease activities, and triphosphate biosynthetic processes (Supplementary Table S13). KEGG functional analysis revealed enrichment in terms related to oxidative phosphorylation and meiosis (Supplementary Table S13). These results suggest that *C. serrulata* has a tendency for cell division and reproduction and undergoes phosphorylation processes. Furthermore, we obtained all types of features of the gene families, including single-copy orthologs, multiple-copy orthologs, other orthologs, and unique genes, present in the related species included in the phylogenetic tree construction (Supplementary Fig. S3b).

**Fig. 4:**
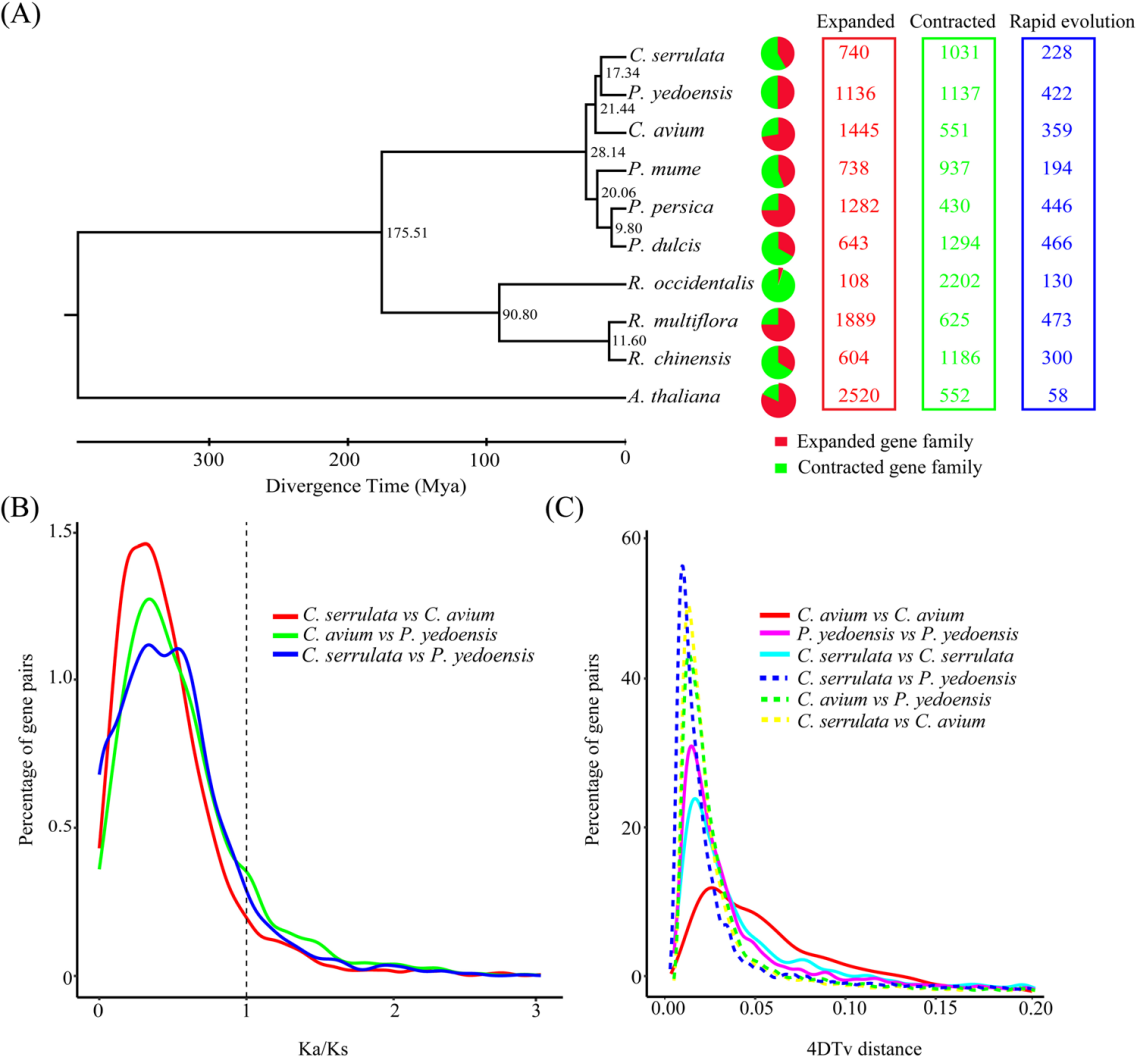
Analysis of the evolution of the *Cerasus serrulata* genome. **a** Phylogenetic tree; divergence time; and profiles of gene families that underwent expansion, contraction, and rapid evolution in ten species. **b** Ka/Ks distribution of paralogs and orthologs of *C. serrulata* compared with that of *C. avium* and *P. yedoensis*. **c** Distribution of 4DTv distances in *C. serrulata*, *C. avium*, and *P. yedoensis*

Seven hundred forty expanded gene families and 1031 contracted gene families were identified by analyzing the differences between the ancestral species and *C. serrulata* using CAFE v4.0 (Fig. 4a and Supplementary Table S14). The expanded and contracted family genes were significantly enriched (*q* < 0.05) in 232 and 266 GO terms of the three categories, respectively (Supplementary Tables S15 and S16). A total of 228 rapidly evolving gene families consisting of 2023 genes were significantly enriched (*q* < 0.01) in 269 GO terms (Supplementary Table S17). The top functional terms involve oxidation–reduction, glycerolipid biosynthetic processes, glycerolipid and lipid metabolism, and so forth. The KEGG enrichment analysis indicated that rapidly evolving genes were relevant to the metabolism of glycine, serine, threonine, phenylalanine, beta-alanine, and tyrosine (Supplementary Fig. S4).

Gene family analysis revealed that 3754 genes were similarly present in all the species, and 656 of these genes were single-copy genes. The phylogenetic tree composed of ten species was generated using 656 single-copy orthologs. The tree indicated that *C. serrulata* was most closely related to *P. yedoensis*, which speciated ~17.34 million years ago (Mya), while the divergence time estimated between *C. serrulata* and *C. avium* was ~21.44 Mya (Fig. 4a). These results demonstrated the close evolutionary relationship between *C. serrulata* and *P. yedoensis*. The phylogenetic tree convincingly showed that the time scale of *Cerasus* and *Prunus* was close, diverging ~28.14 Mya. This verified the taxonomic claim that *Cerasus* is independent of *Prunus*. In addition, the relationship of *Rosa* L., represented by *R. chinensis* and *R. multiflora*, to *Rubus* L., represented by *R. occidentalis*, suggested speciation from ~90.80 Mya. All of the genera shared common ancestors and diverged at ~175.51 Mya. These results are consistent with the observed morphological similarities and differences in the genera.

The homologous gene pairs obtained by MUSCLE were subjected to the KaKs Calculator to calculate each gene pair value. As reflected by the Ka/Ks-density curves of *C. serrulata* vs. *P. yedoensis* and *C. serrulata* vs. *C. avium*, the differences in numbers for the two groups were not large (Fig. 4b). The positively selected genes (Ka/Ks >1) for *C. serrulata* vs. *C. avium* and for *C. serrulata* vs. *P. yedoensis* were composed of 136 and 133 gene pairs, respectively (Supplementary Tables S18 and S19). Transcription factors with definitively matched Pfam domains were identified from these gene pairs and screened to detect their functions. Ten types of transcription factors were found for *C. serrulata* compared to *C. avium*, and eight types were found for *C. serrulata* compared to *P. yedoensis* (Tables 2 and 3). The common transcription factors, including ERF, B3, TCP, bHLH, and NAC types, suggest that these genes are more likely to be positively selected in the process of growth and development, physiological metabolism, and stress responses in *C. serrulata*. The 4DTv values were estimated by comparison with themselves or with each other and indicated that they had experienced only one WGD event (Fig. 4c). Self-comparison peaks at 0.014 (*C. serrulata*), 0.011 (*P. yedoensis*), and 0.028 (*C. avium*) indicated a common WGD event. The peaks of the mutual comparisons appeared slightly earlier than those of the self-comparisons, implying that they had begun to differentiate after the end of the whole-genome replication event. The divergence times of the three species were close: the peak of *C. serrulata* vs. *C. avium* was calculated to be 0.0088, and the peak of *C. serrulata* vs. *P. yedoensis* was calculated to be 0.0069. This suggests that the divergence of *C. avium* occurred earlier than did that of *P. yedoensis*, which is in accordance with the phylogenetic tree results.

**Table 2:** Positively selected genes by *C. serrulata* compared to *C. avium*

Transcription factor	Gene ID	Pfam	Annotation
HD-ZIP	EVM0006705	HD-ZIP_N	Homeobox-leucine zipper protein HAT14
LBD	EVM0013307	Epimerase	Tetraketide alpha-pyrone reductase 2
NAC	EVM0010591	NAM	NAC domain-containing protein 72
M-type_MADS	EVM0017730	RuBisCO_small	Ribulose bisphosphate carboxylase small chain
TCP	EVM0020820	TCP	Transcription factor TCP12
ERF	EVM0025239	AP2	Ethylene-responsive transcription factor protein At4g13040
B3	EVM0026745	B3	B3 domain-containing protein
G2-like	EVM0027330	Myb_DNA binding	Probable transcription factor KAN4
bHLH	EVM0004714	HLH	Transcription factor bHLH62
	EVM0021371	HLH	Transcription factor BIM1
WRKY	EVM0011053	WRKY	WRKY DNA-binding transcription factor 70
	EVM0017221	NB-ARC/LRR_8	Disease-resistance protein At4g27190

**Table 3:** Positively selected genes by *C. serrulata* compared to *P. yedoensis*

Transcription factor	Gene ID	Pfam	Annotation
NAC	EVM0004149	NAM	NAC domain-containing protein 96
TCP	EVM0005613	Cullin	cullin-1
NF-YB	EVM0006103	Lectin_legB	putative L-type lectin domain-containing receptor kinase S.7
TALE	EVM0008637	KNOX2/ELK/Homeobox_KN/KNOX1	homeobox protein knotted-1
bHLH	EVM0008718	HLH	Transcription factor ILR3
MYB	EVM0010759	4HBT	acyl-coenzyme A thioesterase 13
	EVM0016863	Myb_DNA binding	Transcription factor MYB
B3	EVM0011074	B3	B3 domain-containing transcription factor VAL3
	EVM0027593	B3	B3 domain-containing protein REM5
ERF	EVM0017051	GST_C_2/GST_N_3	Probable glutathione S-transferase DHAR2
	EVM0018545	AP2	Dehydration-responsive element-binding protein
	EVM0023070	AP2	AP2/EREBP family transcription factor

### MADS-box gene family in *C. serrulata*

**Fig. 5:**
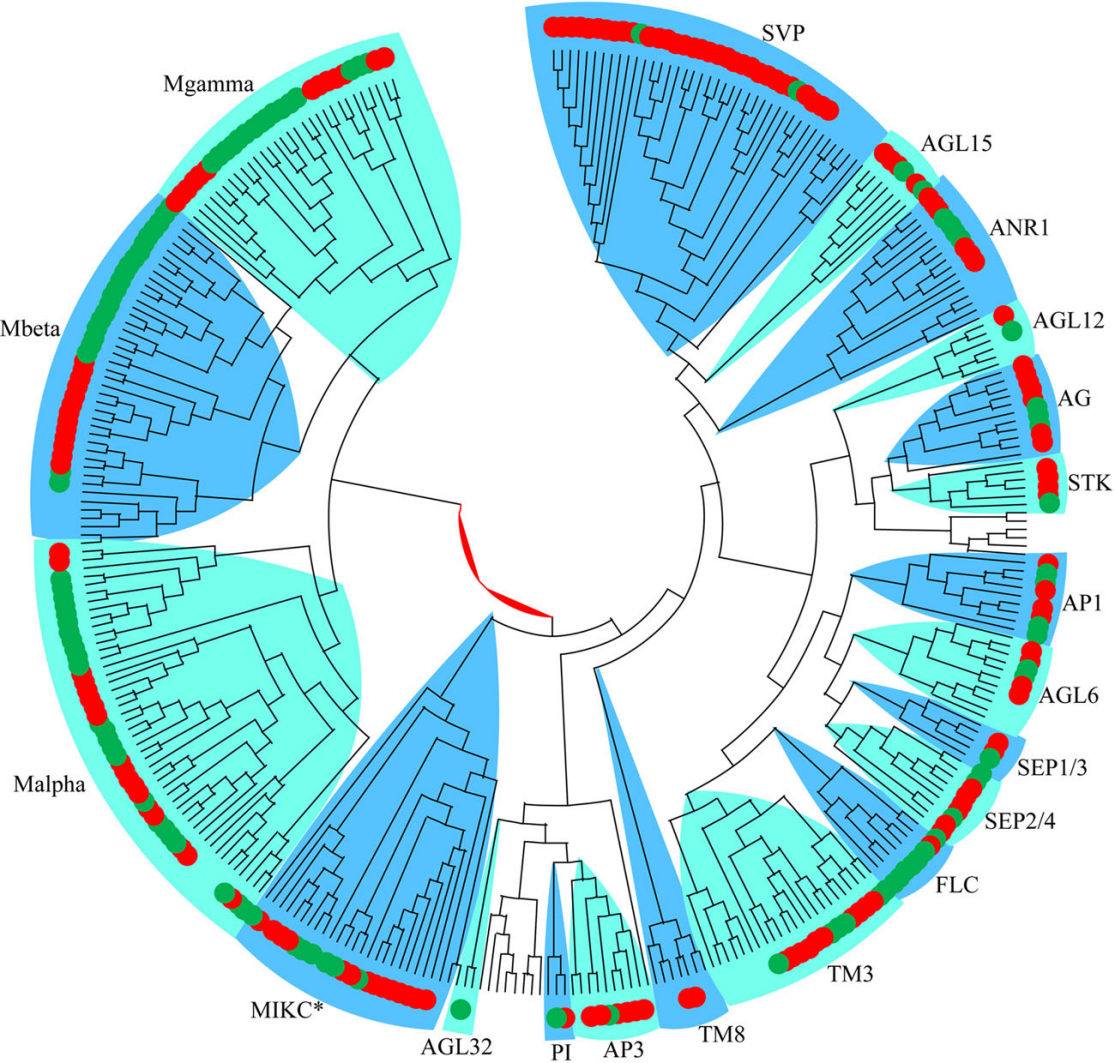
The MADS-box gene family in *Cerasus serrulata* and *Arabidopsis thaliana*. The red color represents genes from *Cerasus serrulata*, and the green color represents genes from *Arabidopsis thaliana*

MADS-box transcription factors control multiple traits in plants and are best known for their regulation of plant floral organ development by type II MIKCc MADS-box genes^23^. The MADS-box gene family has been thoroughly described in Rosaceae genomes, such as those of *P. mume*^20^, *P. persica*^24^, and *M. domestica*^25^. The MADS-box family was identified by aligning to *A. thaliana* using MAFFT 7.0^26^, and the tree was constructed by FastTree v2^27^, with the default parameters. Here, we identified a total of 148 members of MADS-box genes in the genome of *C. serrulata*, including 44 type I MADS-box genes and 104 type II MADS-box genes (Supplementary Table S20). Compared with the number of previously reported MADS-box genes in other Rosaceae species, such as *P. persica* (79) and *M. domestica* (146), the number of MADS-box genes in *C. serrulata* was evidently greater than that in *P. persica*. This suggests that the *C. serrulata* floral organs are relatively well developed, while *P. persica* floral organ development may have been limited by the deletion of some MADS-box genes. The type II MADS-box genes were classified into SVP, AGL15, ANR1, AGL12, AG, STK, AP1, AGL6, SEP1/3, SEP2/4, FLC, TM3, TM8, AP3, and PI subfamilies, in accordance with the classification of *A. thaliana* (Fig. 5). However, we did not detect any members of the AGL32 subfamily in *C. serrulata*, suggesting the loss of several functions that may affect seed development occurred, such as the maternal role in fertilization. In addition, the most expanded subfamily was the SVP subfamily, which expanded to 32 members in *C. serrulata* (there are only four members in *A. thaliana*). Considering that this family plays important roles in early flowering in early spring, we postulate that the expansion of the SVP subfamily is correlated with flowering time control in *C. serrulata*.

### **MYBandWRKYgene** families in *C. serrulata*, *C. avium*, and *P. yedoensis*

**Fig. 6:**
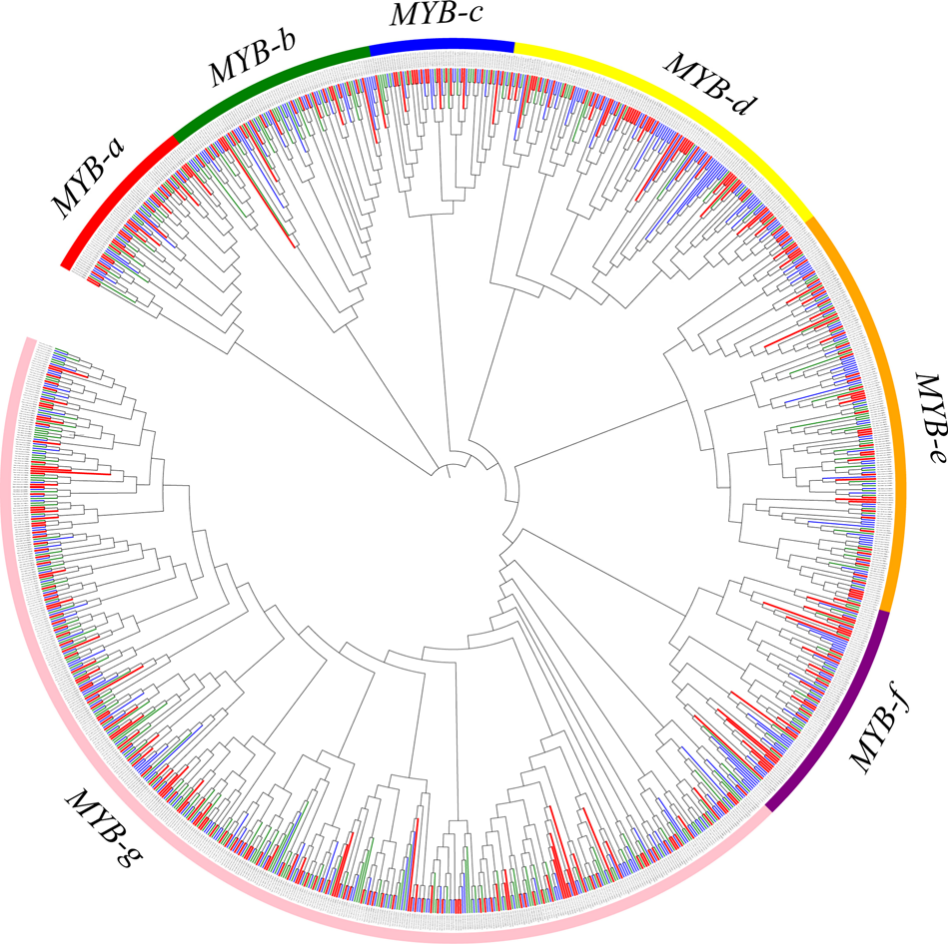
The MYB gene family could be divided into seven subfamilies, namely, subfamilies a–g. The red members represent genes from *C. serrulata*, the blue members represent genes from *C. avium*, and the green members represent genes from *P. yedoensis*

The members of other gene families, such as those of MYB and WRKY transcription factors, play important roles in many biological functions and are widely distributed in plant roots, stems, leaves, and flowers. Members of the MYB and WRKY gene families containing distinct protein domains were identified by alignment to the Pfam and Swiss-Prot database using Pfam and phmmer searches. Phylogenetic trees for the MYB and WRKY gene families were constructed using *C. serrulata*, *C. avium*, and *P. yedoensis* data. The members of the MYB gene family grouped into seven subfamilies (subfamily a–g) containing 1132 genes (Fig. 6). Among 1132 genes, 372 originated from *C. serrulata*, 429 originated from *C. avium*, and 331 originated from *P. yedoensis*. Specifically, subfamily a has 71 members with the same or similar protein domain, and subfamilies b–g have 89, 60, 145, 171, 96, and 500 members, respectively (Supplementary Table S21). For the WRKY gene family, the same method was adopted to ensure the domain and classify the subfamilies. Eight subfamilies (subfamilies a–h) composed of 221 genes were generated and clustered with regularity (Supplementary Fig. S5). Sixty-six genes in *C. serrulata* were present in eight subfamilies, while 77 and 78 genes were found in *C. avium* and *P. yedoensis*, respectively (Supplementary Table S22). The acquisition of a gene family implies species-specific tandem duplication and gene loss events during evolution that may have led to species-specific functional deterioration.

### **Plant disease-resistance genes (**R-genes**) in the Rosaceae**

**Fig. 7:**
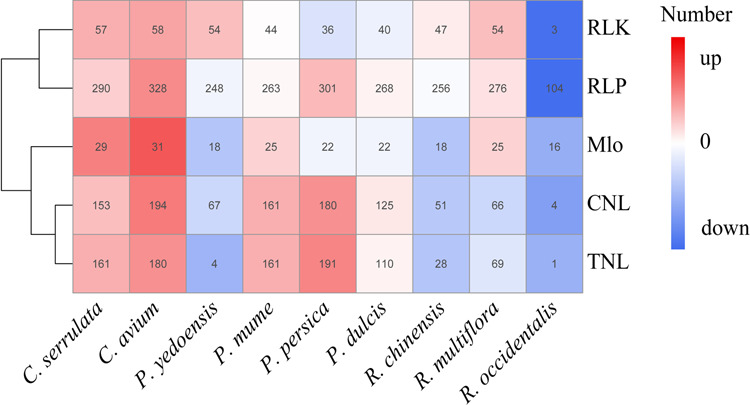
Plant disease-resistance genes (R-genes) identified in nine representative species of *Rosaceae.* The major types, CNLs, TNLs, RLKs, RLPs, and Mlos were identified in nine species

To identify and classify plant disease-resistance genes (R-genes) in Rosaceae species, nine species involved in the gene family analysis were analyzed by aligning to the PRGdb (http://prgdb.crg.eu/wiki/Main_Page) using the BLAST program. In total, 941 R-genes were identified in *C. serrulata*, which was more than that identified in *P. yedoensis* (555) but less than that identified in *C. avium* (1149) (Supplementary Table S23). The common and functionally confirmed R-gene types mainly included five major types: CNLs, TNLs, RLKs, RLPs, and Mlos (Fig. 7). Among all of the types, 161 and 180 TNL R-genes are present in *C. serrulata* and *C. avium*, while only four genes were found in *P. yedoensis*. Of all the representative species, *P. persica* has 1123 R-genes, and *R. occidentalis* has 187 R-genes, which are two species with the most and the least R-genes, respectively. For many plants, resistance is considered one of the most important traits that is controlled by R-genes and is affected by the environment. Our results provide fundamental information on the R-genes in these species, which are a prerequisite for resistance.

## Discussion

The complete genomes of *C. avium* and *P. yedoensis* were previously sequenced and published^12,19^. As an important germplasm resource of *Cerasus*, *C. serrulata* is cultivated worldwide for its beautiful blossoms and superior ornamental characteristics. Resolving the genome sequence of *C. serrulata* is thus of great significance. Genome assembly can be relatively challenging due to high heterozygosity percentages and high proportions of repeat sequences. However, with the development of long-read sequencing techniques, the assemblies of heterozygous genomes have been largely successful^28–30^. In this work, we de novo assembled the *C. serrulata* genome with a chromosomal-level assembly using a combination of Nanopore and Hi-C sequencing techniques. The assembled genome is 265.4 Mb in length, which is smaller than both the *C. avium* genome (272.4 Mb) and *P. yedoensis* genome (323.8 Mb). The contig and scaffold N50 values of 1.56 and 31.12 Mb, respectively, are higher than those of *C. avium* (scaffold N50 = 291.57 kb) and *P. yedoensis* (contig N50 = 132.59 kb, scaffold N50 = 198.95 kb). These results demonstrate that a high-quality *C. serrulata* genome was generated and could serve as a credible whole-genome reference sequence for research into the flowering cherry.

The *magnum opus* of Linnaeus, Species Plantarum, included the initial classification of *Prunus*, which included cherry, plum, peach, plum, apricot, and so on^31^. The earliest taxonomic history of *Cerasus* can be traced back to 1753, and *Cerasus* was suggested to be independent of *Prunus* in 1754^32^. Since then, the parallel existence of *Prunus* and *Cerasus* has been accepted. However, the application of molecular techniques has indicated that the *Cerasus* subgenus should belong to a single genus^4^. The *C. serrulata* genome and the comparative analysis of related species suggest that *P. yedoensis* should be classified as *C. yedoensis*. Moreover, the phylogenetic tree demonstrated that *C. serrulata*, *C. avium*, and *P. yedoensis* clustered together with the shortest divergence time and were distinct from other species of *Prunus*. The relatively recent differentiation of *C. serrulata* and *P. yedoensis* suggests that they are evolutionarily closely related. 4DTv analysis revealed that *C. serrulata*, *C. avium*, and *P. yedoensis* experienced one common WGD event and that the divergence of *C. avium* occurred earlier than that of *P. yedoensis*. Thus, *C. serrulata* and *P. yedoensis* diverged relatively recently, while *C. avium* diverged from the ancestor of these two species much earlier.

The colinear map revealed a high chromosome-level assembly of the *C. serrulata* genome in comparison with the *P. persica* genome. The colinear map of the *C. serrulata* genome and *C. avium* genome showed that they had many identical sequences on different chromosomes, evidently more than that in the colinear map of *C. serrulata* and *P. persica*. A previous analysis based on a phylogenetic tree and 4DTv analysis convincingly demonstrated that the evolutionary relationship of *C. serrulata* and *C. avium* was closer than that of *C. serrulata* and *P. persica*. We postulate that, even though there are differences between the *C. serrulata* and *C. avium* genomes, they do not exceed the differences between the *C. serrulata* and *P. persica* genomes. The only reasonable explanation is that there could be assembly errors in the genomes of *C. serrulata* and *C. avium*. The assembly quality of *C. serrulata* was verified in several ways, including via BUSCO, the mapping ratio for Illumina, and the colinear map of *C. serrulata* and *P. persica*. However, further evidence or experiments are still needed to confirm this.

*Cerasus serrulata* is cultivated for its flowers, while *C. avium* is cultivated for its fruit; thus, an assessment of the characteristics of its genomes is warranted. Based on synteny analysis, we discovered 333 syntenic blocks sharing 14,072 syntenic genes. Among these genes, eight chromosomes containing 12,397 genes demonstrated a one-to-one correspondence. No large-scale chromosome translocation or chromosome rearrangement was observed between the two genomes, implying that *C. serrulata* is relatively evolutionarily conserved. In addition, we studied the commonness and intricacies of *C. serrulata*, *C. avium*, and *P. yedoensis*, including their gene families, positively selected genes, and plant disease-resistance genes (R-genes), and focused on the identification and clustering of the MYB and WRKY family genes in particular. We enriched specific family genes and positively selected genes to determine the key aspects of differentiating *C. serrulata*. The results showed that *P. yedoensis* had only 554 R-genes, while *C. avium* and *P. yedoensis* had 1149 and 941 R-genes, respectively. Among the five major types, the number of type TNL R-genes differed significantly, and *C. serrulata*, *C. avium*, and *P. yedoensis* were found to have 161, 180, and four TNL R-genes, respectively. *Prunus yedoensis* is a hybrid of *P. speciosa* and *P. subhirtella*, and is an important cultivar for ornamental purposes^33,34^. This remarkable contrast illustrates that many R-genes for *P. yedoensis* have been lost, such that its ability to survive in a harsh environment may decrease during the process of artificial selection and hybridization. R-genes play essential roles in breeding processes, and their acquisition enables us to determine on which resistant traits different types of R-genes govern.

Chinese flowering cherry is an important ornamental tree species during early spring and is widely planted in gardens. Understanding the regulation of floral organ development will help improve the varieties and explain the diversity in flowering cherry. The MADS-box gene family is known to be related to floral organ development. We identified 148 MADS-box members in the genome of *C. serrulata*; these members included 44 type I MADS-box genes and 104 type II MADS-box genes. Within the type II MADS-box genes, the loss of the AGL32 subfamily might affect seed development. The SVP subfamily expanded to 32 members in *C. serrulata*, whereas only four members disappeared in *A. thaliana*. The functions of the SVP subfamily members are related to early flowering, and the expansion event implies that these members may influence or control flowering time. Our results presented here may provide new insights into the current problems associated with genetic diversity and offer valuable information for evolutionary studies on *C. serrulata* and other *Cerasus* species.

## Materials and methods

### Plant materials and extraction of DNA

*C. serrulata* trees were grown in Baohua Provincial Nature Reserve, Jurong, Jiangsu (119.05 E, 32.06 N, 240 m above sea level). Fresh leaves and other tissues were collected for the deposition of specimens at the Herbarium of Nanjing Forestry University (specimen number: YXG18032501) and for sequencing at Biomarker Technologies Corporation, Beijing. High-quality genomic DNA was extracted from the leaves using a DNeasy Plant Mini Kit (Qiagen, Germany). The concentration and purity of the extracted DNA were assessed using a Nanodrop 2000 spectrophotometer (Thermo, MA, USA) and Qubit 3.0 (Thermo, CA, USA), and the integrity of the DNA was measured using pulsed-field electrophoresis with 0.8% agarose gel.

### Library preparation and sequencing

A short-read library was prepared with a Nextera XT Library Prep Kit (Illumina, CA, USA). Approximately 1 μg of genomic DNA was extracted for short-read library construction and sheared to short fragment sizes of ~350 bp using a Covaris S2 sonicator (Covaris, MA, USA). The qualified library was sequenced on an Illumina X-ten platform (Illumina, CA, USA) with a paired-end 150 (PE150) read layout. In total, 38.14 Gb of clean data (~148.62 × the assembled genome) were generated and used for genome survey and correction.

Approximately 10 μg of genomic DNA was prepared for long-read library construction using an ONT Template Prep Kit (Nanopore, Oxford, UK). According to the manufacturer’s instructions, the total DNA was sheared to sizes of ~20 kb, and the sheared fragments were recovered by a BluePippin™ System using the processes of damage repair, end repair, and blunt-end adapter ligation via a NEBNext FFPE DNA Repair Mix Kit. The qualified library was subsequently sequenced on the Nanopore PromethION platform with an R9 cell sequencing reagent kit.

For Hi-C library construction, samples were digested with a restriction enzyme (HindIII), in situ labeled with a biotinylated residue, and end repaired. Purified DNA was sheared to a length of 300–700 bp using a NEBNext Ultra II DNA Library Prep Kit (Illumina). The remaining data consisted of 122.84 million clean reads (~36.77 Gb) following the removal of low-quality reads.

Four tissues (leaves, buds, flowers, and roots) collected from the same *C. serrulata* tree were mixed together for transcriptome sequencing. All of the Illumina and Nanopore sequencing data were deposited in the SRA database under NCBI BioProject ID PRJNA596558. The assembly and annotation data that supported the findings of this study have been deposited in the Figshare database, accessible via the following respective URLs: 10.6084/m9.figshare.12431846.v3 and 10.6084/m9.figshare.12431864.v3.

### Estimation of genomic features

Short reads for Illumina were filtered by Fastp v0.19.3^35^, after which they were randomly selected and prepared for contamination assessment using BLAST v2.2.31^36^ with an *E*-value = 1e^−05^. We estimated overall genomic features, namely, size, heterozygosity, and repeats, by plotting the 19-mer depth distribution (*k* = 19) using Jellyfish v2^37^. In brief, the average *k*-mer depth was divided by the total *k*-mer numbers to calculate the genomic features according to the formula. Repetitive sequences were estimated where the depth of the *k*-mer was more than two times that of the main peak, and heterozygous sequences were estimated where the depth was half of the main peak.

### Genome assembly

The original offline data in FAST5 format obtained from the Nanopore PromethION platform were converted into fastq format using the Guppy procedure embedded in MinKNOW (Oxford Nanopore). Long reads that were low in quality and with short fragments (minimum length cutoff of 2000 bp) were filtered and removed using Fastp v0.19.3^35^. Primary assemblies of the Nanopore long reads were mainly performed by Canu v1.5^38^ and WTDBG v1.1^39^, with adjusted parameters. Long reads were assembled into genomic contigs automatically using Canu v1.5^38^, with the following parameters: genomeSize = 300 m and corOutCoverage = 100. SMARTdenovo v1.0^40^ was the third tool used for the assembly. The results of the assemblies were efficiently merged using Quickmerge v0.2.2^41^, and the redundant data were removed with Numer v4.0.0^42^. The consensus of the merged assembly as input was polished and corrected three times using Racon^30^. Moreover, Illumina reads specifically for genome feature estimation were prepared for the correction of the final assembly using Pilon v1.22^43^, which also performed three corrections.

### Hi-C scaffolding

An assembled genome can be perfectly identified by chromatin interactions using the Hi-C technique. A total of 122.84 million clean reads were mapped to the primary assembly by Nanopore long reads using BWA aligner v0.7.10-r789^44^ with the default parameters. In cases where both ends of the paired reads mapped to the assembly, only reads of the two pairs that were uniquely mapped were used for further analysis. There were two different types of mapped paired reads obtained by assessing self-circle ligation, dangling ends, and religation: valid interaction pairs and invalid interaction pairs. To obtain valid interactive Hi-C reads, reads of self-ligation, nonligation, dangling ends, and other invalid reads were filtered and removed using Hi-C-Pro v2.10.0^45^.

The contigs of the primary assembly were broken into 50-kb fragments normalized by the restriction sites. Candidate chromosomes were then generated automatically, and the reassembled contigs were divided into ordered, oriented, and anchored groups using Lachesis v2e27abb^46^ with the following parameters: CLUSTER_MIN_RE_SITES = 37, CLUSTER_MAX_LINK_DENSITY = 2, CLUSTER_NONINFORMATIVE_RATIO = 2, ORDER_MIN_N_RES_IN_TRUN = 33, and ORDER_MIN_N_RES_IN_SHREDS = 31. To further improve the assembly of Hi-C, gaps in the Hi-C assembly were filled using LR GapCloser v1.1^47^.

### Genome-quality evaluation

BUSCO v4.0.6^48^ and CEGMA v2.5^49^ are two tools frequently used to evaluate the level of final genome completeness. The Illumina reads for the genome survey were mapped to the final assembled genome using SAMtools v0.1.18^50^. Considering that it had low conservativeness in interspecies repeat sequences, it was necessary to construct a specific repetitive sequence database of *C. serrulata* for repeat predictions. Repetitive sequences of the *C. serrulata* genome were first predicted using LTR FINDER v1.05^51^, RepeatScout v1.0.5^52^, and PILER-DF v2.4^53^, and DNA repeats were identified and classified using PASTEClassifier v1.0^54^. When the predictions and Repbase results were combined, a final repeat database for only *C. serrulata* was generated using RepeatMasker v4.0.7^55^.

### Genome annotation

Protein-coding gene prediction was performed using three classic strategies: ab initio prediction, homology-based prediction, and transcriptome-based prediction. For ab initio prediction, five tools, GenScan v3.1^56^, Augustus v3.1^57^, GlimmerHMM v1.2^58^, Gene ID v1.4^59^, and SNAP v2006-07-28^60^, were used to predict the coding genes with model training by turn. For homology-based prediction, protein sequences from five representative species (*Arabidopsis thaliana*^61^, *Prunus persica*^13^, *Malus domestica*^14^, *Prunus dulcis*^15^, and *Rubus occidentalis*^18^) were downloaded from databases and aligned to the *C. serrulata* protein sequences using GeMoMa v1.3.1^62^. For transcriptome-based prediction, transcriptome sequencing data obtained in a previous study were used for predicting genes using HISAT v2.0.4^63^, StringTie v1.2.3^63^, TransDecoder v2.0^64^, and GeneMark v5.1^65^ according to the respective workflows. Three classic prediction strategies were subsequently integrated into nonredundant protein-coding genes using EVM v1.1.1^66^ and then modified by PASA v2.0.2^67^.

Pseudogenes and noncoding RNAs, including microRNA, ribosomal RNA (rRNA), and transfer RNA (tRNA), were identified and assessed during this step. In the process of searching for putative pseudogenes, they were assessed based on the premature stop codons or frameshift mutations within the structure of genes using GenBlastA v1.0.4^68^ and GeneWise v2.4.1^69^. MicroRNA and rRNA were identified by Infernal 1.1^69^ based on information from miRBase^70^ and Rfam^71^, respectively, and the tRNA was identified using tRNAscan-SE v1.3.1^72^.

Based on the sequence similarity and domain conservation, protein-coding genes were annotated by aligning to the EuKaryotic Orthologous Groups (KOG)^73^, Kyoto Encyclopedia of Genes and Genomes (KEGG)^74^, TrEMBL^75^, Swiss-Prot^75^, and Nonredundant (Nr) databases^76^ using BLAST v2.2.31^36^, with a maximal E-value of 1e^−05^. Additionally, functional genes were identified and searched by aligning to the Pfam database^77^ sequences and Gene Ontology (GO) terms^78^ using HMMER V3.0^79^ and the BLAST2GO v2.5 pipeline^80^, respectively.

### Synteny analysis

*Cerasus* species can be divided into those that are cultivated for their flowers and those that are cultivated for their fruit, as represented by *C. serrulata* and *C. avium* (*Cerasus avium*), respectively. The genome of *P. persica* (*Prunus persica*) has been widely used for its high-quality assembly. In this analysis, the genome sequence of two species with a chromosomal-level assembly, those of *P. persica* (*Prunus* L.)^13^ and *C. avium*^12^, were downloaded from databases and compared with the genome sequence of *C. serrulata*. The final chromosomes of *C. serrulata* were considered to be arranged in the best order by referring to the recognized *P. persica* genome. For collinearity analysis, we compared the *C. serrulata* genome with the genomes of *P. persica* and *C. avium* using MUMmer (http://mummer.sourceforge.net), with the parameter l = 10,000. In addition, the genomes of *C. serrulata* and *C. avium* were subjected to a synteny analysis to reveal syntenic blocks in detail and the associated genes using BLASTP (*E* < 1e^−05^)^36^. Each syntenic block comprising at least five sequential genes with no obvious error is displayed in the synteny map.

### Gene family identification

To identify gene families from the protein-coding genes, protein sequences from *C. serrulata* and other representative species, including *C. avium*^12^, *P. persica*^13^, *P. dulcis* (*Prunus dulcis*)^15^, *P. mume* (*Prunus mume*)^20^, *P. yedoensis* (*Prunus yedoensis*)^19^, *R. chinensis* (*Rosa chinensis*)^21^, *R. multiflora* (*Rosa multiflora*)^22^, and *R. occidentalis* (*Rubus occidentalis*)^18^, were compared with each other using BLASTP, with a maximal E-value of 10^−05^. Markov chain clustering was performed by all-to-all analysis. The proteins predicted from all species with sequence lengths >100 amino acids were queried against the Pfam database using Pfam scan^77^. The profiles of gene family expansion, contraction, and rapid evolution were analyzed by comparing the differences between the ancestor and each species using CAFE v4.0^81^. Each gene module was extracted, and its contents were subjected to GO and KEGG functional enrichment analyses. There are usually multiple transcripts present for one gene, and only the longest transcript of each gene obtained from all of the species was considered a single-copy ortholog. The orthologous and paralogous genes of the protein datasets were condensed by removing redundancy via OrthoMCL v2.0.9^82^.

### Phylogenetic analysis

Protein alignments with gaps removed for each single-copy gene family were acquired using the programs MAFFT^83^ and trimAL^84^. The best substitution model for the alignment was estimated using ModelFinder^85^, with the default settings. The single-copy orthologous genes generated were aligned to a superalignment matrix with the guidance of protein alignment using MUSCLE 3.8.31^86^. A phylogenetic tree comprising *C. serrulata* and eight related species, *C. avium*^12^, *P. persica*^13^, *P. dulcis*^15^, *P. mume*^20^, *P. yedoensis*^19^, *R. chinensis*^21^, *R. multiflora*^22^, and *R. occidentalis*^18^, was constructed using PhyML v3.0^87^ with the default parameters and *A. thaliana*^61^ as an outgroup. The recommended divergence time from a fossil was obtained from the TimeTree database, and a molecular clock was employed to date the divergence event using PAML v4.8^88^ with the approximate likelihood calculation method.

### Whole-genome duplication (WGD) analysis

In general, the ratio of the nonsynonymous substitution rate (*K*a) and the synonymous substitution rate (*K*s) was used to assess gene selection. Protein sequences of homologous genes from *C. serrulata* vs. those of representative species were aligned using MUSCLE^86^. As input files, the sequences of the homologous genes were imported into the KaKs Calculator to calculate the gene pair values. Positively selected genes in which *K*a/*K*s > 1 were subsequently selected. In addition, CDS alignments obtained from previous protein sequence alignments were used to detect WGD based on the 4DTv values using the HKY model.

## Supplementary information

Additional files

Table S8 Integrated lists of gene annotation for Cerasus serrulata genome

Table S9 The family of miRNAs, rRNAs and tRNAs in Cerasus serrulata

Table S12 Specific family genes annotated in Cerasus serrulata

Table S14 Expanded and contracted gene families in Cerrasus serrulata

Table S15 Expanded genes in Cerasus serrulata

Table S16 Contracted genes in Cerasus serrulata

Table S17 GO enrichment of rapidly evolving genes in Cerasus serrulata

Table S18 Positively selected gene pairs annotated in Cerasus serrulata and Cerasus avium

Table S19 Positively selected gene pairs annotated in Cerasus serrulata and Prunus yedoensis

Table S20 MADS-box family genes in Cerasus serrulata

Table S21 MYB family genes in Cerasus serrulata, Cerasus avium and Prunus yedoensis

Table S22 WRKY family genes in Cerasus serrulata, Cerasus avium and Prunus yedoensis

Table S23 Detail information for plant disease resistance genes in representative species of Rosaceae
