# Supplementary material for: The genome of Chinese flowering cherry (Cerasus serrulata) provides new insights into Cerasus species
Source: Hortic Res. 2020 Oct 1;7:165. doi: 10.1038/s41438-020-00382-1 (PMC7527954; doi:10.1038/s41438-020-00382-1)
Supplement: Supplementary file 1 — Additional files [file 41438_2020_382_MOESM1_ESM.docx]

**Table S1** Statistics of the length distribution of Nanopore Subreads

| Length (bp) | Number | Total length (bp) | Average length (bp) | Percent (%) |
| --- | --- | --- | --- | --- |
| 2,000~5,000 | 122,442 | 434,327,563 | 3,547.21 | 0.90 |
| 5,000~10,000 | 201,670 | 1,473,234,693 | 7,305.17 | 3.06 |
| 10,000~20,000 | 435,354 | 6,824,729,941 | 15,676.27 | 14.22 |
| 20,000~30,000 | 530,479 | 13,017,282,665 | 24,538.73 | 27.12 |
| 30,000~40,000 | 287,880 | 9,928,778,151 | 34,489.29 | 20.68 |
| 40,000~50,000 | 154,091 | 6,851,000,290 | 44,460.74 | 14.27 |
| 50,000~60,000 | 80,025 | 4,355,663,409 | 54,428.78 | 9.07 |
| 60,000~70,000 | 39,498 | 2,542,900,882 | 64,380.49 | 5.29 |
| 70,000~80,000 | 18,243 | 1,355,697,055 | 74,313.27 | 2.82 |
| >=80,000 | 13,143 | 1,207,324,090 | 91,860.61 | 2.51 |

**Table S2** Statistics of the different types of Hi-C reads

| Mapping Type | | Ratio to the primary assembly (%) | Ratio to unique mapped read pairs (%) |
| --- | --- | --- | --- |
| Total Read Pairs | | 122,843,407 (100) | - |
| Mapped Reads Pairs | | 95,719,582 (77.92) | - |
| Unique Mapped Read Pairs | | 53,349,154 (43.43) | 53,349,154 (100) |
| Valid Interaction Pairs | | 41,199,012 (33.54) | 41,199,012 (77.23) |
| Invalid Interaction Pairs | Dangling End Pairs | 12,150,142 (9.89) | 3327370 (6.24) |
|  | Re-ligation Pairs |  | 2895072 (5.43) |
|  | Self-cycle Pairs |  | 515212 (0.97) |
|  | Dumped Pairs |  | 5412488 (10.15) |

**Table S3** Summary of the Hi-C assembly

| Group | Cluster number | Cluster length (bp) | Order number | Order length (bp) |
| --- | --- | --- | --- | --- |
| Chr01 | 54 | 52,247,480 | 43 | 49,822,012 |
| Chr02 | 39 | 35,145,258 | 29 | 32,766,916 |
| Chr03 | 42 | 35,183,451 | 35 | 33,346,824 |
| Chr04 | 34 | 31,445,416 | 33 | 31,116,561 |
| Chr05 | 37 | 28,918,376 | 31 | 27,176,319 |
| Chr06 | 25 | 27,300,701 | 23 | 27,118,509 |
| Chr07 | 28 | 26,435,496 | 24 | 25,594,122 |
| Chr08 | 30 | 26,480,342 | 27 | 25,308,010 |
| Total (Ratio %) | 289 (95.07) | 263,156,520 (99.16) | 245 (84.78) | 252,249,273 (95.86) |

**Table S4** Summary of the assessment of genome assembly

| Parameter | BUSCO groups (%) | CEGMA groups (%) | Illumina reads ratio (%) |
| --- | --- | --- | --- |
| Total BUSCOs | 1,614 (100) | - | - |
| Complete BUSCOs | 1,528 (94.67) | - | - |
| Complete and single-copy BUSCOs | 1,352 (83.77) | - | - |
| Complete and duplicated BUSCOs | 176 (10.90) | - | - |
| Fragmented BUSCOs | 30 (1.86) | - | - |
| Missing BUSCOs | 56 (3.47) | - | - |
| Total CEGMA | - | 458 (100) | - |
| Searched CEGMA | - | 447 (97.60) | - |
| Number of 248 highly conserved | - | 239 (52.18) | - |
| Total Reads | - | - | 259,743,657 (100) |
| Mapped Reads | - | - | 254,769,545 (98.08) |
| Mapped Reads pairs | - | - | 230,618,535 (88.79) |

**Table S5** Statistics of the repeated sequences

| Type | Number | Length（bp） | Percentage (%) |
| --- | --- | --- | --- |
| ClassI | 263,087 | 100,136,046 | 37.73 |
| ClassI/DIRS | 3,900 | 2,700,117 | 1.02 |
| ClassI/LARD | 120,518 | 35,778,969 | 13.48 |
| ClassI/LINE | 11,808 | 3,662,034 | 1.38 |
| ClassI/LTR/Copia | 48,020 | 25,272,646 | 9.52 |
| ClassI/LTR/Gypsy | 63,885 | 34,234,831 | 12.9 |
| ClassI/LTR/Unknown | 12,849 | 3,696,888 | 1.39 |
| ClassI/PLE | 1,277 | 726,386 | 0.27 |
| ClassI/SINE | 1 | 76 | 0 |
| ClassI/TRIM | 798 | 1,216,391 | 0.46 |
| ClassI/Unknown | 31 | 5,026 | 0 |
| ClassII | 74,021 | 28,488,536 | 10.73 |
| ClassII/Crypton | 5 | 443 | 0 |
| ClassII/Helitron | 15,201 | 4,672,273 | 1.76 |
| ClassII/Maverick | 19 | 14,087 | 0.01 |
| ClassII/TIR | 50,450 | 22,062,606 | 8.31 |
| ClassII/Unknown | 8,346 | 2,029,109 | 0.76 |
| Potential Host Gene | 17,241 | 4,371,180 | 1.65 |
| Unknown | 20,867 | 5,901,526 | 2.22 |
| Total | 375,216 | 130,112,934 | 49.02 |

**Table S6** Summary of the gene prediction results

| Method | Software | Species | Gene number |
| --- | --- | --- | --- |
| Ab initio | Genscan | - | 19,739 |
|  | Augustus | - | 21,461 |
|  | GlimmerHMM | - | 31,031 |
|  | GeneID | - | 30,785 |
|  | SNAP | - | 32,328 |
| Homology-based | GeMoMa | *Arabidopsis thaliana* | 23,430 |
|  |  | *Malus domestica* | 27,376 |
|  |  | *Prunus dulcis* | 36,786 |
|  |  | *Prunus persica* | 32,593 |
|  |  | *Rubus occidentalis* | 25,021 |
| Transcriptome-based | PASA | - | 63,421 |
|  | GeneMarkS-T | - | 36,655 |
|  | TransDecoder | - | 25,246 |
| Integration | EVM | - | 29,094 |

**Table S7** Statistics of gene annotation to different databases

| Annotation database | Annotated number | Percentage (%) |
| --- | --- | --- |
| GO_Annotation | 12,834 | 44.11 |
| KEGG_Annotation | 9,249 | 31.79 |
| KOG_Annotation | 14,363 | 49.37 |
| TrEMBL_Annotation | 27,189 | 93.45 |
| Pfam_Annotation | 24,574 | 88.46 |
| Swiss prot_Annotation | 11,107 | 38.18 |
| Nr_Annotation | 27,589 | 94.83 |
| All_Annotated | 27,611 | 94.90 |

**Table S10** Syntenic blocks between *Cerasus serrulata* and *Cerasus avium*

| C.serrulata  C.avium | C^e^01 | C^e^02 | C^e^03 | C^e^04 | C^e^05 | C^e^06 | C^e^07 | C^e^08 |
| --- | --- | --- | --- | --- | --- | --- | --- | --- |
| C^a^01 | 31 | 1 | 12 | 0 | 5 | 7 | 4 | 5 |
| C^a^02 | 2 | 29 | 1 | 0 | 8 | 10 | 0 | 1 |
| C^a^03 | 5 | 0 | 20 | 3 | 4 | 0 | 5 | 0 |
| C^a^04 | 2 | 1 | 5 | 32 | 1 | 0 | 0 | 0 |
| C^a^05 | 6 | 7 | 6 | 0 | 20 | 0 | 0 | 0 |
| C^a^06 | 1 | 9 | 0 | 1 | 0 | 23 | 1 | 1 |
| C^a^07 | 6 | 1 | 4 | 0 | 1 | 2 | 20 | 1 |
| C^a^08 | 5 | 1 | 0 | 0 | 0 | 2 | 3 | 18 |
| Total | 58 | 49 | 48 | 36 | 39 | 44 | 33 | 26 |

C^e^01-08: The 8 chromosomes of *Cerasus serrulata*; C^a^01-08: The 8 chromosomes of *Cerasus avium.*

**Table S11** Genes involved in the syntenic blocks between *Cerasus serrulata* and *Cerasus avium*

| 1. serrulata   C. avium | C^e^01 | C^e^02 | C^e^03 | C^e^04 | C^e^05 | C^e^06 | C^e^07 | C^e^08 |
| --- | --- | --- | --- | --- | --- | --- | --- | --- |
| C^a^01 | 2737 | 60 | 56 | 71 | 69 | 17 | 64 | 62 |
| C^a^02 | 14 | 1386 | 0 | 33 | 64 | 90 | 30 | 15 |
| C^a^03 | 102 | 58 | 1392 | 51 | 60 | 0 | 52 | 0 |
| C^a^04 | 0 | 0 | 23 | 1357 | 0 | 14 | 0 | 0 |
| C^a^05 | 88 | 84 | 44 | 21 | 1312 | 0 | 9 | 0 |
| C^a^06 | 56 | 112 | 0 | 0 | 0 | 1706 | 16 | 16 |
| C^a^07 | 55 | 0 | 69 | 0 | 0 | 9 | 1267 | 19 |
| C^a^08 | 50 | 8 | 0 | 0 | 0 | 7 | 7 | 1240 |
| Total | 3102 | 1708 | 1584 | 1533 | 1505 | 1843 | 1445 | 1352 |

C^e^01-08: The 8 chromosomes of *Cerasus serrulata*; C^a^01-08: The 8 chromosomes of *Cerasus avium*.

**Table S13** Significantly enriched GO and KEGG terms for specific family genes

| GO_ID | GO_Terms | GO_Class | Adjusted *P*-value |
| --- | --- | --- | --- |
| GO:0004523 | RNA-DNA hybrid ribonuclease activity | MF | 1.60E-06 |
| GO:0016891 | endoribonuclease activity | MF | 1.60E-06 |
| GO:0003676 | nucleic acid binding | MF | 1.04E-05 |
| GO:0140098 | catalytic activity, acting on RNA | MF | 1.33E-05 |
| GO:0016787 | hydrolase activity | MF | 1.33E-05 |
| GO:0097159 | organic cyclic compound binding | MF | 1.70E-05 |
| GO:0004540 | ribonuclease activity | MF | 3.82E-05 |
| GO:0004518 | nuclease activity | MF | 3.82E-05 |
| GO:0015074 | DNA integration | BP | 0.0002842 |
| GO:0015002 | heme-copper terminal oxidase activity | MF | 0.003186 |
| GO:0008324 | cation transmembrane transporter activity | MF | 0.0066375 |
| GO:0003954 | NADH dehydrogenase activity | MF | 0.0066375 |
| GO:0098655 | cation transmembrane transport | BP | 0.009744 |
| GO:0006754 | ATP biosynthetic process | BP | 0.009744 |
| GO:0009145 | purine nucleoside triphosphate biosynthe | BP | 0.012744 |
| GO:0009142 | nucleoside triphosphate biosynthetic process | BP | 0.036744 |
| ko00190 | Oxidative phosphorylation | - | 0.001012088 |
| ko04113 | Meiosis | - | 0.008809388 |

BP: Biological process; MF: Molecular function; CC: Cellular component.
